# Supplementary figures and images for: Fatty acids and chlorogenic acid content in Plectranthus edulis root tubers
Source: PLoS One. 2024 Jul 8;19(7):e0305910. doi: 10.1371/journal.pone.0305910 (PMC11230528; doi:10.1371/journal.pone.0305910)

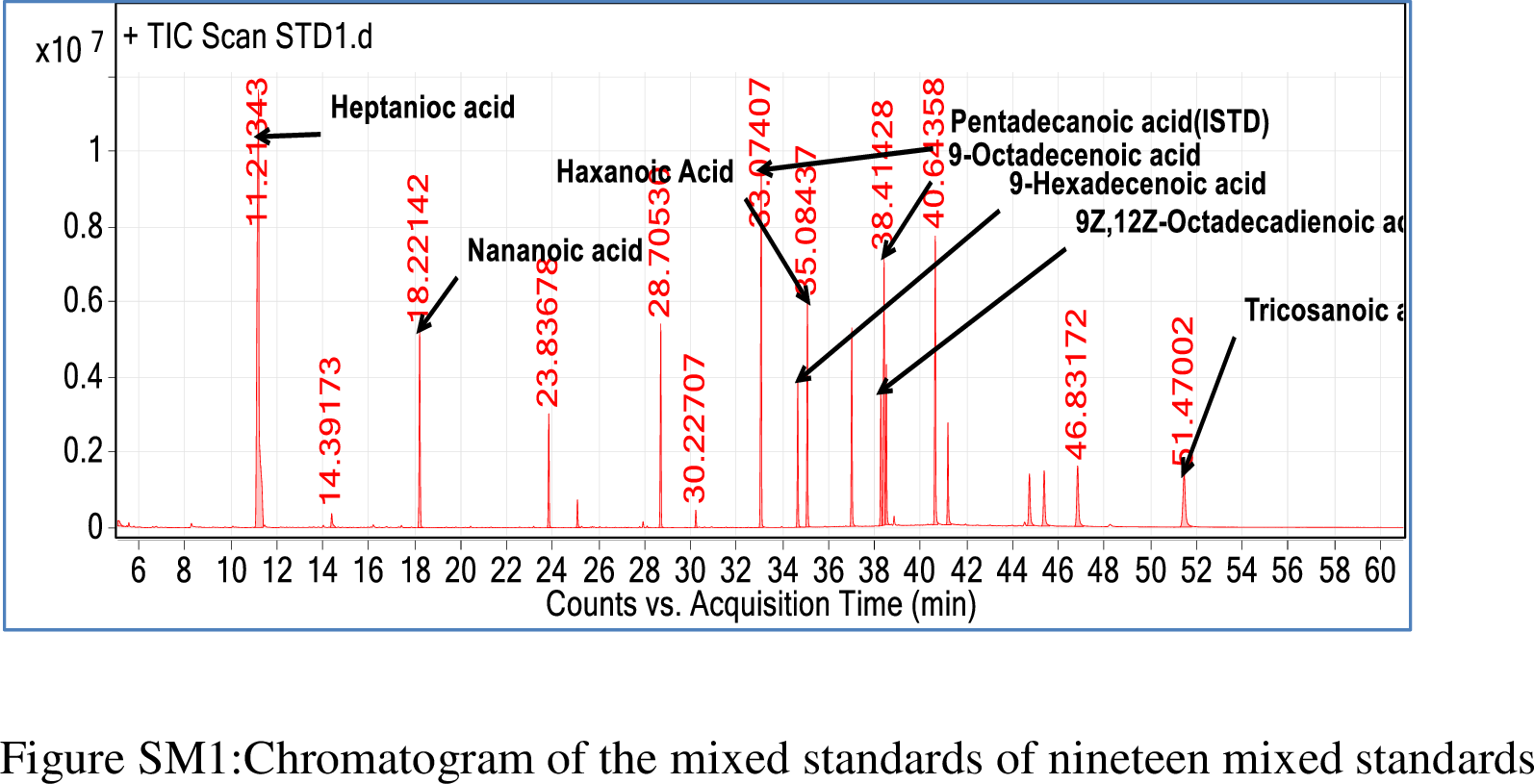

Supplement: S1 Fig — (TIF) [file pone.0305910.s001.tif]
