# Supplementary material for: Fatty acids and chlorogenic acid content in Plectranthus edulis root tubers
Source: PLoS One. 2024 Jul 8;19(7):e0305910. doi: 10.1371/journal.pone.0305910 (PMC11230528; doi:10.1371/journal.pone.0305910)
Supplement: S1 Table — (DOCX) [file pone.0305910.s002.docx]

Table SM1: Crude fat content of *P.edulis*

| No | Kebele | Mas of sample (g) | Mass of the crude oil (g) | %Crude fat |
| --- | --- | --- | --- | --- |
| 1 | Lemma | 1 | 0.0647 | 6.47% |
| 2 | Gambisi | 1 | 0.0553 | 5.53% |
| 3 | Betsena | 1 | 0.0569 | 5.69% |
| 4 | Dangiya | 1 | 0.0533 | 5.33% |
| 5 | Zengena | 1 | 0.0504 | 5.04% |
